# Coronavirus Disease 2019 (COVID-19): A Pediatric Perspective

**DOI:** 10.31729/jnma.4977

**Published:** 2020-07-31

**Authors:** Rupesh Shrestha, Laxman Shrestha

**Affiliations:** 1 Department of Pediatrics, Tribhuvan University Teaching Hospital, Maharajgunj, Kathmandu, Nepal

**Keywords:** *coronavirus*, *pregnancy outcome*, *vertical infectious disease transmission*

## Abstract

Coronavirus disease 2019, the new public health emergency that originated in China, is spreading rapidly across the globe with limited tools to confine this growing pandemic. The virus, severe acute respiratory syndrome coronavirus 2, is transmitted by droplet infection from person to person. Our current understanding of the disease spectrum is limited. The proportion of infected children is significantly less compared to adults with the majority of them showing mild symptoms. More than half of symptomatic children present with fever and cough. However, the extent of asymptomatic infection in children and the role they play in community transmission is still undetermined. Although there are case reports of neonates infected with severe acute respiratory syndrome coronavirus 2, vertical transmission from infected mother to new-born is yet to be proven. The disease is confirmed by demonstration of the virus by real-time reverse transcriptase-polymerase chain reaction in respiratory secretions. Due to the lack of specific antiviral agents, we rely on infection-control measures to prevent disease spread and on supportive care for infected ones. This article has summarized the clinical characteristics of children with coronavirus disease 2019 based on published case reports.

## INTRODUCTION

Coronavirus disease 2019 (COVID-19) is a respiratory disease caused by severe acute respiratory syndrome coronavirus 2 (SARS-CoV-2), also referred to as the 2019 novel coronavirus (2019-nCoV), with a high incidence of pneumonia in infected individuals. After it's outbreak in Wuhan City of China in December 2019, it has rapidly spread to the rest of the world and the World Health Organization (WHO) has declared it as a pandemic on March 11, 2020. Our understanding of COVID-19 is evolving along with increasing numbers of cases. SARS-CoV-2 is a newly emerging contagious pathogen without pre-existing immunity in humans. Available data show that the effects on children are less severe than those on adults. Most of the pediatric cases are family cluster cases having epidemiological links to adults.^1^ Two coronaviruses causing severe illness in humans have occurred in the past 2 decades: severe acute respiratory syndrome (SARS) in 2003 and Middle East respiratory syndrome (MERS) in 2012.

## ETIOLOGY

SARS-CoV-2 belongs to the ß-coronavirus genus.^23^ Coronaviruses (CoVs) are enveloped, positive-stranded Ribonucleic Acid (RNA) viruses with crownlike appearance under an electron microscope due to the presence of spike glycoprotein.^3,4^ CoVs can be divided into four genera: alpha, beta, delta, and gamma, of which alpha and beta CoVs are known to infect humans.^2,5^ Till date, seven human coronaviruses (HCoVs) have been identified i.e., HCoV 229E, NL63, OC43, and HKU1, SARS-CoV, MERS-CoV, and SARS-CoV-2. The first four coronaviruses are endemic globally and account for 10% to 30% of self-limiting upper respiratory tract infections in immunocompetent individuals.^3^ SARS-CoV-2 is sensitive to ultraviolet rays and heat, and can be effectively inactivated by lipid solvents including ether (75%), ethanol (60-90%), chlorine-containing disinfectant, peroxyacetic acid, and chloroform except for chlorhexidine.^3^ The virus has been shown to use the angiotensin-converting enzyme 2 (ACE2) for cell entry similar to the severe acute respiratory syndrome (SARS) coronavirus.^6^

Genomic analyses suggested that the SARS-CoV-2 probably originated from bats as the virus had 89% to 96% genomic identity with bat SARS-like coronavirus and was transmitted to humans by some other intermediary host.^7–9^ The intermediate host may have a role in causing recombination and mutation in the virus with the expansion of genetic diversity. Researchers in China identified the pangolin as one of the potential sources of SARS-CoV-2 because genetic sequences of viruses isolated from the scaly animals were 99% similar to that of the circulating virus.^8^ Later, Xia hypothesized that SARS-CoV-2 could have evolved in canid mammalian intestine or tissues associated with the intestine.^10^ However, the actual origin of the virus remains unclear.

## PATHOGENESIS

At first, animal to human transmission was assumed to be the main mechanism of disease spread as the initial cases were linked to the direct exposure to the Huanan seafood wholesale market of Wuhan. Subsequent cases were not associated with this exposure. Person to person transmission of the disease has been confirmed by the studies.^11,12^ Infection is transmitted by inhalation of large droplets generated during coughing, talking, or sneezing by both symptomatic and asymptomatic persons within close range (within six feet). Infection can also be acquired by touching surfaces contaminated by these droplets and then touching the nose, mouth, and eyes.^4^ Transmission through aerosols can also occur under specific circumstances such as tracheal intubation and extubation, noninvasive ventilation, manual ventilation before intubation, cardiopulmonary resuscitation, bronchoscopy, administration of high-flow oxygen or nebulized medications, tracheotomy and upper gastrointestinal endoscopy.^13^ The infected droplets can spread one to two meters and deposit on surfaces where it remains viable for a variable period. The virus was detectable up to four hours on copper, up to 24 hours on cardboard, and up to two to three days on plastic and stainless steel surfaces.^14^ The airborne spread has not been reported so far.

The virus has been detected in specimens like bronchoalveolar lavage fluid, sputum, nasal swabs, pharyngeal swabs, feces, and blood with decreasing frequency.^12,15^ The viral load detected in the nasal swab of the asymptomatic patient was similar to that in the symptomatic patients.^16^ However, the viral load in nasopharyngeal swabs can vary according to the disease severity, severe cases having significantly higher (~60 times) load than that of mild cases.^17^ The study done by Xu Y, et al. failed to demonstrate the replication-competent virus in fecal swabs, which is required to confirm the potential for fecal-oral transmission.^18^ On contrary to this, live SARS-CoV-2 has been detected in a stool sample of a few patients.^15^ According to a joint WHO-China report, the fecal-oral transmission did not appear to be a significant factor in the spread of infection.^9^ Hence, the fecal-oral route of transmission is yet to be established. Viral shedding in the respiratory specimen is also found to be longer in children with COVID-19.^12^ Prolonged fecal and nasal secretion of the virus in children has substantial implications for community spread.

## MATERNAL TO FETAL TRANSMISSION

Controversy exists regarding whether SARS-CoV-2 can be transmitted in utero from an infected mother to her infant before birth. In one study analyzing 38 pregnant women with COVID-19 and their newborn in China, there were no confirmed cases of intrauterine transmission of SARS-CoV-2 from mothers to their fetuses.^19^ All the neonatal specimen tested including some placentas were negative for the virus. However, Zeng, et al. reported early onset SARS-CoV-2 infection in three out of 33 neonates born to mothers with COVID-19.^20^ All three neonates tested positive for the virus in nasopharyngeal and anal swabs and had chest radiologic image of pneumonia. The vertical maternal to the fetal transmission of the disease could not be ruled out in a given cohort as the source of SARS-CoV-2 in the neonates could be of maternal origin. Likewise, there are case reports of three neonates with elevated SARS-CoV-2 immunoglobulin M (IgM) in the blood collected within few hours of birth who were born to mother with COVID-19.^21,22^ The elevated IgM antibodies suggested that the neonates were infected in utero. However, nasopharyngeal swabs of all three neonates were negative by RT-PCR. Additional examination of maternal and newborn samples should be done to confirm this preliminary observation.

It is unknown whether SARS-CoV-2 can be transmitted through breast milk. The report of testing breast milk samples of six mothers with COVID-19 found no virus in the maternal milk.^23^ Newborns may still acquire a SARS-CoV-2 infection through close contact with infected mothers. Thus, mothers with confirmed or suspected COVID-19 should take all precautions to prevent transmission to the infant during feeding. Considering the benefits of breastfeeding and the insignificant role of breastmilk in the transmission of other respiratory viruses, United Nations Children's Fund (UNICEF) has recommended continuing with breastfeeding while applying necessary precautions to prevent transmission of infection.

## CLINICAL FEATURES

The incubation period of 2019-nCoV infections ranges from one to fourteen days.^2^ People are thought to be most contagious when they are most symptomatic. Symptoms of COVID-19 are varied and the disease presentation can range from no symptoms (asymptomatic) to severe pneumonia with multi-organ dysfunction and death.^4,9^ A significant proportion of children with COVID-19 do not appear to develop any symptom, or have subclinical symptoms. Relatively few children are hospitalized, and fewer children than adults experience fever, cough or shortness of breath, according to the report from the Centers for disease control and prevention (CDC). Among pediatric patients, children under age one year, and children with underlying health conditions are at the highest risk of serious illness resulting in hospitalization.^24^ Chronic lung disease (including asthma), cardiovascular disease, and immunosuppression are common underlying health conditions in children with COVID-19. Clinical features in symptomatic children tend to be mild and they recover within one to two weeks after the onset of illness.^2,8,25^

Children of all ages are susceptible to COVID-19 including neonates with no significant gender difference. However, young children, particularly infants, were vulnerable to SARS-CoV-2 infection.^8^ Dong, et al.^8^ reported the proportion of severe and critical cases for the age groups <1, 1-5, 6-10, 11-15, and >15 years as 10.6%, 7.3%, 4.2%, 4.1%, and 3.0% respectively. The most common presenting symptoms include fever followed by cough in more than half of symptomatic children. Upper respiratory tract symptoms such as rhinorrhea and sore throat are also relatively common and it is not uncommon for children to have diarrhea and/or vomiting, even in some cases as their sole presenting features (Table 1).

**Table 1:** Clinical features of children with COVID-19.

First Author	Total cases	Cough	Fever	Sore throat	Rhinorrhea	Vomiting	Diarrhea	Asymptomatic	Abnormal chest imaging
**Cai**^12^	**10**	**6**	**7**	**4**	**2**	**0**	**0**	**0**	**4**
Chen^26^	31	12	14	2	2	0	2	12	11
Xia^27^	20	13	12	1	3	2	3	0	16
Liu^28^	6	6	6	6	1	4	0	0	4
Wei^29^	9	1	4	0	1	0	0	1	^*^
Xu^18^	10	5	6	4	2	0	2	1	5
Zhang^30^	34	20	26	0	0	4	4	0	28
Lu^31^	171	83	71	79	13	11	15	27	111
Qui^32^	36	7	13	2	0	2	2	10	19
Wang^33^	31	14	20	2	2	2	3	4	14
Feng^34^	15	1	5	0	1	0	0	8	12
Zhu^35^	10	3	4	0	0	0	0	3	5
Han^36^	7	5	5	1	0	4	4	0	5
CDC^24^	291	158	163	71	21	31	37	0	^*^
Total	681	334	356	172	48	60	72	66	234

* : information not provided.

From available data, it is not possible to determine the extent of infection among children, since many asymptomatic children are unlikely to be tested, and the role they play in the transmission of disease. People interviewed by the Joint Mission Team could not recall episodes in which transmission occurred from a child to an adult.^9^ However, if children are infected but asymptomatic, they could serve as a source of transmission to adults.^37^

Data on individuals aged 18 years old and under suggest that there is a relatively low attack rate in this age group. Separate studies were done in Italy, China, and United States (US) including 22,512, 72,314 and 149,082 cases respectively showed the incidence of 1.2%, 2%, and 1.7% among patients aged <18 years respectively.^24,38–9^ The lower than expected rates of COVID-19 infection in children might be due to decreased exposure to the virus and or patients who are sick, decreased infection with the virus because of immunity to other respiratory viruses including coronaviruses, or decreased likelihood of illness, even when infected with the virus.^8,37^ Incompletely developed a child's immune system may respond to the pathogen differently than the adult immune system.^8^ Children also appear to be relatively spared of severe disease. Mild illness in children may be related to trained immunity.^25^

Trained immunity refers to the use of certain vaccines such as Bacille Calmette-Guerin (BCG) to train innate immunity to generate immune memory. It is speculated that children were less sensitive to the virus because the maturity and function of ACE2, receptor for SARS-CoV-2 in children may be lower than that in adults.^40^

The largest review of children with COVID-19 to date including 2143 children in China reported severe cases (defined as hypoxia) in 5.6% children and critical cases like acute respiratory distress syndrome (ARDS)/multiorgan dysfunction syndrome in 0.6% children.^8^ The proportion of severe and critical cases was highest in the age group of less than one year. There are only a few reported deaths among children due to COVID-19.

### LABORATORY FINDING

The white blood cell counts can vary in children with COVID-19. Lymphopenia and leukocytosis are relatively uncommon in children, with raised lymphocyte counts and diminished total white cell count present in 17-22% of children (Table 2).

**Table 2:** Total white cell count and lymphocyte count in children with COVID-19.

First author	Total cases	Total white cell count		Lymphocyte	
		Leukopenia	Leukocytosis	lymphopenia	Lymphocytosis
Xia^27^	20	4	2	7	3
Zhang^30^	34	0	0	0	17
Qui^32^	36	7	0	1	0
Wang^33^	31	2	3	2	4
Cai^12^	10	1	0	0	1
Chen^26^	31	12	1	0	17
Feng^34^	15	8	0	0	0
Zhu^35^	10	0	0	0	1
Han^36^	7	0	2	0	0
Total	194	34	8	10	43

These findings are different from adults where lymphopenia appears most common.^41–43^ Inflammatory markers such as C-reactive protein (CRP) and procalcitonin, liver transaminases, and D-dimers are often raised only very mildly in children and are not common in children as compared with the adults.

## RADIOLOGICAL FEATURES

Radiologic features in children also differ from their adult counterparts. Based on published case reports on children with COVID-19, radiological evidence of pneumonia is present in up to two-thirds of children irrespective of symptoms (Table 1). Changes may be present even in asymptomatic children. When present computed tomography (CT) chest abnormalities are often less severe. Common CT chest features include ground-glass opacities and patchy shadows in the outer lung fields, mainly in the subpleural area (Table 3).

**Table 3:** Chest CT scan finding in children with COVID-19.

Total	First author	CT Chest finding cases	No. of patients
Xu^18^	10	Ground-glass opacities in outer lung fields	5
Chen^26^	31	Ground-glass opacities in subpleural field	11
		Subpleural lesions	20
Xia^27^	20	Ground-glass opacities in subpleural field	12
		Consolidation with surrounding halo	10
Liu^28^	6	Ground-glass opacities	1
		Patchy shadows	3
Zhang^30^	34	Patchy lesions in lung lobules	33
		Ground-glass opacities	1
Lu^31^	171	Ground-glass opacities	77
		Patchy shadows	53
Qui^32^	36	Ground-glass opacities	19
Wang^33^	31	Patchy ground-glass opacities in the middle and outer zones of lung and near the pleural area	9
		Abnormal finding not specified	5
Feng^34^	15	Ground-glass opacities	10
		Patchy shadows	2
Li^44^	5	Patchy ground-glass opacities	3
Zhu^35^	10	Ground glass opacity	5
Total	369		

Lesions were bilateral in up to two-thirds of cases with abnormal CT scans. Fifty percent of chest CT scans had consolidation with a surrounding halo in one case series.^27^ Lung consolidation may occur in severe cases.^2^

## DIAGNOSIS

Any suspected case should be tested for the infection with SARS-CoV-2 using a molecular test. A Suspect case is: i) a patient with acute respiratory illness, and a history of travel to or residence in a location reporting community transmission of COVID-19 disease during the 14 days before symptom onset; or ii) a patient with an acute respiratory illness and having been in contact with a confirmed or probable COVID-19 case in the last 14 days before symptom onset, or iii) a patient with severe acute respiratory illness, and requiring hospitalization, and in the absence of an alternative diagnosis that fully explains the clinical presentation.^45^ These definitions are based on the currently available information and are regularly revised as new information accumulates.

The CDC recommends collection of a nasopharyngeal swabs to test for SARS-CoV-2.^46^ Alternative specimens include oropharyngeal swabs, sputum (if produced), and endotracheal aspirate or bronchoalveolar lavage from patients who are intubated. SARS-CoV-2 RNA is detected by reverse-transcription polymerase chain reaction (RT-PCR), a positive test of which confirms the diagnosis. If a negative result is obtained from a patient with a high index of suspicion for COVID-19, particularly when only upper respiratory tract specimens were collected, additional specimens, including from the lower respiratory tract if possible, should be collected and tested.^47^

## MANAGEMENT

Treatment is essentially supportive and symptomatic depending upon the severity of the disease. Neither antiviral agent nor immunomodulatory therapy with exact efficacy is available till now to be used in children with COVID-19. Antiviral therapy (oseltamivir, ganciclovir, and lopinavir/ritonavir), interferon-D, corticosteroids, and intravenous immunoglobulin have been used to treat children.^4,26,28,30,32^ Several investigational agents are being explored for the treatment of COVID-19. These include redemsivir (a nucleotide analog with in-vitro activity against SARS-CoV-2), interleukin-6 pathway inhibitors (tocilizumab, siltuximab, sarilumab), convalescent plasma, favipiravir (an RNA polymerase inhibitor), and chloroquine/hydroxychloroquine (antimalarials) among many others. More evidence is needed before these drugs are recommended.

In hypoxic patients, the provision of oxygen through nasal prongs, face mask, high flow nasal cannula, or non-invasive ventilation is indicated. Antibiotics and antifungals are required if co-infections are suspected or proven. Management of severe cases is similar to the management of most viral pneumonia-causing respiratory failure.^48^ Mechanical ventilation may be needed in patients with ARDS. Evidence-based treatment guidelines for ARDS should be followed that includes conservative fluid strategies, empirical early antibiotics for suspected bacterial co-infection, lung-protective ventilation, prone ventilation, and consideration of extracorporeal membrane oxygenation for refractory hypoxemia. Septic shock and specific organ dysfunction appear to occur in a significant proportion of patients with COVID-1 9-related critical illness and are associated with increasing mortality.^48^

COVID-19 pneumonia is thought to be a specific condition characterized by severe hypoxemia and nearly normal lung compliance with two phenotypes: Type L (Low elastance, Low ventilation-to-perfusion ratio, Low lung weight, and Low recruit ability) and Type H (High elastance, High right-to-left shunt, High lung weight, and High recruit ability).^49^ Type L patients can be ventilated with higher volumes (6-9 ml/kg of tidal volume) without the risk of ventilator-induced lung injury due to high lung compliance whereas type H patients are treated the same as ARDS.

An epidemiological study has shown that countries without universal BCG vaccination have been severely affected both in terms of mortality and spread of infection compared to countries with universal and longstanding BCG policies.^50^ It may be related to a positive non-specific immune response like increased secretion of pro-inflammatory cytokines like IL-1B triggered by the BCG vaccination that has antiviral immunity.

In the absence of specific therapy directed against the virus, it is of paramount importance to implement infection control practices by controlling the source of infection, blocking the route of transmission, and protecting the susceptible population. Prevention involves isolation of suspected and confirmed cases, and strict infection control measures at hospitals that include contact and droplet precautions. Non-specific manifestations of the disease, infection transmission both before the onset of symptoms and after clinical recovery, long incubation period, tropism for mucosal surface, and prolonged duration of the illness makes the infection prevention and control measures very challenging. Appropriate social distancing and daily preventive behaviors are of utmost importance for all age groups.

COVID-19 is a novel disease with an incompletely described clinical course, especially in children. Children are mainly infected with SARS-CoV-2 from their family members but less severely than adults, presenting mild symptoms, and a good prognosis. It is necessary to learn as much as possible through observational studies and clinical trials across a wide range of patients, populations, and health care settings.

## Conflict of Interest

**None.**
